# Assessment of Binding Interaction between Bovine Lactoferrin and Tetracycline Hydrochloride: Multi-Spectroscopic Analyses and Molecular Modeling

**DOI:** 10.3390/molecules23081900

**Published:** 2018-07-30

**Authors:** Qifan Sun, Xin Gao, Hongna Bi, Yingbo Xie, Lin Tang

**Affiliations:** College of Life Sciences, Shandong Provincial Key Laboratory of Animal Resistance Biology, Shandong Normal University, Jinan 250014, China; sunqifansdnu@163.com (Q.S.); gaoxinsdnu2015@163.com (X.G.); bihongna2010@163.com (H.B.); shanshitl@163.com (Y.X.)

**Keywords:** bovine lactoferrin, tetracycline hydrochloride, mutil-spectral technique, microscale thermophoresis, molecular docking

## Abstract

In this paper, the interaction between bovine lactoferrin (bLf) and tetracycline hydrochloride (TCH) was researched by microscale thermophoresis (MST), multi-spectroscopic methods, and molecular docking techniques. Normal fluorescence results showed that TCH effectively quenched the intrinsic fluorescence of bLf via static quenching. Moreover, MST confirmed that the combination force between bLf and TCH was very strong. Thermodynamic parameters and molecular docking further revealed that electrostatic forces, van der Waals, and hydrogen bonding forces played vital roles in the interaction between bLf and TCH. The binding distance and energy transfer efficiency between TCH and bLf were 2.81 nm and 0.053, respectively. Moreover, the results of circular dichroism spectra (CD), ultraviolet visible (UV-vis) absorption spectra, fluorescence Excitation-Emission Matrix (EEM) spectra, and molecular docking verified bLf indeed combined with TCH, and caused the changes of conformation of bLf. The influence of TCH on the functional changes of the protein was studied through the analysis of the change of the bLf surface hydrophobicity and research of the binding forces between bLf and iron ion. These results indicated that change in the structure and function of bLf were due to the interaction between bLf and TCH.

## 1. Introduction

**Figure 1:**
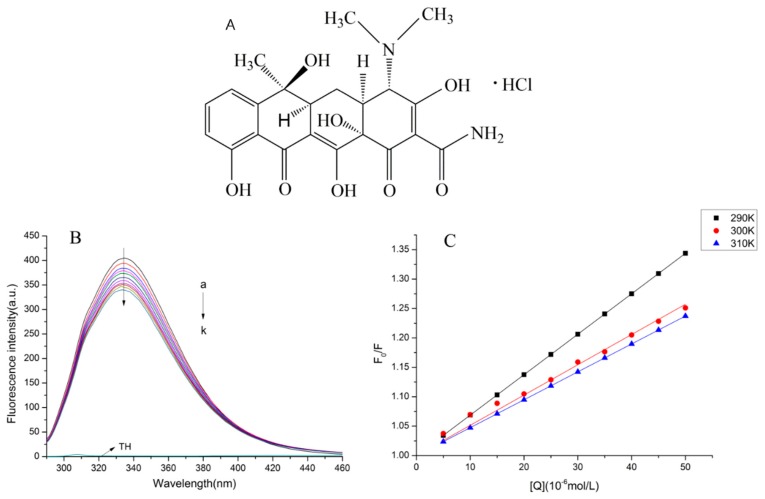
(**A**) The chemical structure of tetracycline hydrochloride (TCH). (**B**) Effect of TCH on fluorescence spectra of bovine lactoferrin (bLf). (**C**) The Stern–Volmer plots for the quenching of bLf by TCH at different temperatures.

Tetracycline hydrochloride (TCH, as shown at Figure 1A), an important class of tetracycline antibiotics, has become a widely used veterinary medicine for therapeutic and prophylactic purposes [1]. It has been seen as one of the most effective drugs for the treatment of dairy cow mastitis [2]. However, if the dosage is poorly controlled, it will cause residues of tetracycline in animal or animal products [3]. As a very popular nutrient, milk consumption is increasing year by year with the improvement of people’s living standards [4]. Although it can provide people with rich protein, it is not clear whether the remaining antibiotics in milk have affected the structure and function of milk proteins, and whether the effects will cause harm to humans. Therefore, it is important to study the effect of antibiotics residues in milk on bovine proteins.

As an abundant whey protein in milk, Bovine lactoferrin (bLf) has multi-biological functions such as antibacterial, antiviral, antifungal, anti-carcinogenic, and anti-inflammatory activity [5,6]. It consists of a single polypeptide chain with about 708 amino acids and the molecular weight is about 80 kDa [7]. The tertiary structure of bLf has two iron-binding sites (N- and C-lobes), giving it the capability to bind with two Fe^3+^ ions [8,9]. Moreover, Kanyshkova et al. found that some functional properties of bLf are related to its iron binding capacity [10].

Additionally, Chen et al. have studied the interaction between lomefloxacin and bLf, and they found that the binding of lomefloxacin influenced the structure of bLf to a certain extent [11]. Yang et al. researched the molecular interaction between bLf and EGCG, and the conclusion was that EGCG made the bLf structure more compact by making changes to the tertiary structure [12]. However, there is no research studying the effects of tetracycline on structural and conformational changes of bLf until now.

In this paper, we measured the interaction between TCH and bLf and analyzed the structural and functional changes of bLf. We hope the results of the paper can help us understand the importance of controlling the dosage of antibiotics in the dairy industry and for food safety [13,14].

## 2. Results

### 2.1. Normal Fluorescence Spectra

Figure 1B revealed that the fluorescence intensity of bLf was quenched regularly with an increasing concentration of TCH. The total quenching fluorescence intensity was arrived at around 80 a.u. Moreover, the maximum emission wavelength of bLf was changed from 332.2 nm to 333.6 nm. The change was negligible.

### 2.2. The Fluorescence Quenching Mechanism

The fluorescence quenching is usually divided into static quenching and dynamic quenching. Static quenching is controlled by forming a complex substance. Therefore, with the increase of temperature, the static quenching constant will decrease due to the lower stability of the complex. Excited fluorescence quencher molecules colliding with quencher is called dynamic quenching. They are usually analyzed according to the equation of Stern–Volmer as follows [15]:

*F*_0_/*F* = 1 + *K_q_τ*_0_ [*Q*] = 1 + *K_SV_* [*Q*] (1)

where *F*_0_ and *F* are the fluorescence intensities in the absence and presence of ligand, [*Q*], *K_SV_*, *K_q_* and *τ*_0_ are the ligand concentration, Stern–Volmer quenching constant, quenching rate constant, and average lifetime of the biomolecule without ligand (*τ*_0_ = 10^−8^ s), respectively [16].

The presented work aimed to investigate whether TCH interacts with bLf and to further confirm the quenching mechanism. In order to confirm the quenching mechanism, we analyzed the fluorescence data by the Stern–Volmer equation at different temperatures (290 K, 300 K, and 310 K). The results were shown in Figure 1C and Table 1.

Figure 1C and Table 1 showed that the *K_SV_* of bLf decreased with increasing temperature and the Value of *K_q_* were much higher than 2.0 × 10^10^ L/mol/s (the maximum diffusion collision quenching constant value) [17].

### 2.3. Binding Parameters and Thermodynamic Parameters

In order to calculate the binding site and binding constant, the binding equation described by Scatchard was used in this paper [18,19]:

log[(*F*_0_ ‒ *F*)/*F*] = log*K_a_* + *n*log[*Q*] (2)

where *F*_0_, *F*, and [*Q*] are the same as in Equation (1), *K_a_* is the binding constant, and *n* is the number of binding sites.

The interaction forces between ligand and biomolecule usually include hydrogen bonding, van der Waals forces, electrostatic forces, and hydrophobic interaction forces [20]. Since the temperature effect is very small, the enthalpy change (Δ*H*) and the entropy change (Δ*S*) can be regarded as the constants to deduce the type of non-covalent bond forces. In this paper, Δ*H*, Δ*G*, and Δ*S* between bLf and TCH were calculated based on the van’t Hoff equation (Equation (3)) and thermodynamic equation (Equation (4)) [21,22]:

(3) $$\ln K_{a}=-\Delta H/RT\;+\;\Delta S/R$$

(4) $$\Delta G=\Delta H-T\Delta S=-RT\;\ln K_{a},$$

where *R* is the universal gas constant (8.314 J mol^−1^ K^−1^); *T* is the experimental temperature. The calculated thermodynamic parameters and *K_a_* values for the binding interaction between TCH and bLf are listed in Table 2.

### 2.4. Energy Transfer between bLf and TCH

The distance between the donor and the acceptor and the energy transfer efficiency can be determined by the following formula [23,24,25]:

(5) $$E=1-\frac{F}{F_{0}}\;=\;\frac{R_{0}^{6}}{R_{0}^{6}+r^{6}}$$

where *r* is the distance between the acceptor and the donor, *R*_0_ is the critical distance when the transfer efficiency is 50%, which can be calculated by:

*R*_0_^6^ = 8.79 × 10^−25^ [*K*^2^*n*^−4^*ΦJ*(*λ*)] (6)

where *K*^2^ is the spatial orientation factor of the dipole, *n* represents the refractive index of the medium, *Φ* represents the fluorescence quantum yield of the donor, and *J* represents the overlap integral of the fluorescence emission spectrum of the donor and the absorption spectrum of the acceptor:

(7) $$J(\lambda )=\frac{\sum F\left(\lambda \right)\varepsilon \left(\lambda \right)\lambda^{4}\Delta \lambda }{\sum F\left(\lambda \right)\Delta \lambda }$$

where *F(λ)* is the fluorescence intensity of the fluorescence donor at wavelength *λ* and *ε*(*λ*) is the molar absorption coefficient of the acceptor at wavelength *λ*.

**Figure 2:**
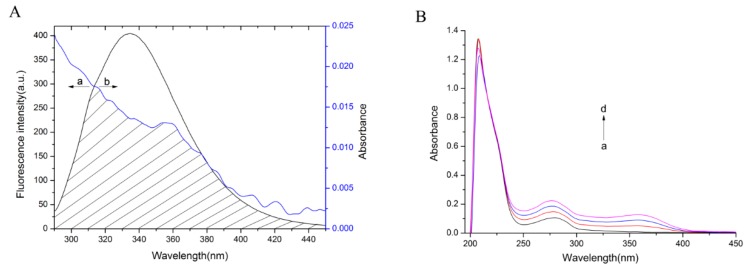
(**A**) Spectral overlaps of the fluorescence spectra of bLf with the absorption spectra of TCH. (**B**) UV-vis absorption spectra of bLf without and with TCH.

The overlap of the absorption spectrum of TCH and the fluorescence emission spectrum of bLf was shown in Figure 2A and the values were shown in Table 3.

### 2.5. BLf-TCH Interaction Studies by Microscale Thermophoresis

**Figure 3:**
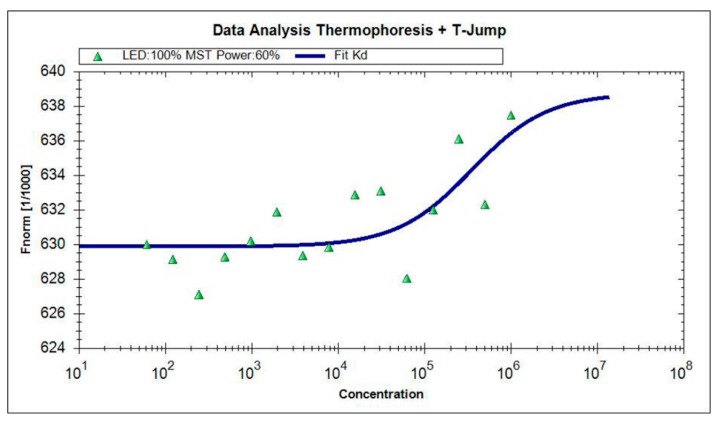
Competitive experiments by MST. The binding of TCH to a constant amount of bLf was quantified. The final bLf was labeled, and TCH was added at varying concentrations (ranging from 122.08 nM to 2 mM).

MST was utilized to further prove the interactions results by fluorescence spectroscopy. The titration curve fitting by MST was shown in Figure 3. The equilibrium dissociation constant *K_d_* value computation formula is as follows:

*K_d_* = [A][B]/[AB] (8)

[AB] is the concentration of the complex at the time of equilibrium, [A] and [B] are the concentration of free A and B at equilibrium. In Equation (8), A represents bLf, B represents TCH. The value of *K_d_* represents the dissociation degree of bLf-TCH complex at the equilibrium state. The larger the *K_d_* value, the more dissociation, the weaker the affinity between bLf-TCH; on the contrary, the lower the *K_d_* value, the affinity is stronger between bLf-TCH.

### 2.6. Effect of the TCH on the bLf Conformation

#### 2.6.1. Conformational Investigation by UV-vis Absorption Spectra

The UV-vis absorption spectra were measured to explore the structural change of bLf with the addition of TCH. Figure 2B clearly showed that the absorption intensity of bLf increased and a new absorption peak appeared at 360 nm with gradual addition of TCH to bLf solution. We speculated that the new absorption peaks are the peak of the newly generated structures. Furthermore, Figure 2B revealed that the maximum peak positions of the complexes had a slight red shift.

#### 2.6.2. Conformational Investigation by Synchronous Fluorescence

The synchronous fluorescence spectra can provide information about the molecular environment in the vicinity of chromospheres. When the wavelength intervals (∆*λ*) were stabilized at 15 or 60 nm, the synchronous fluorescence gave the characteristic information of tyrosine or tryptophan residues, respectively [26].

**Figure 4:**
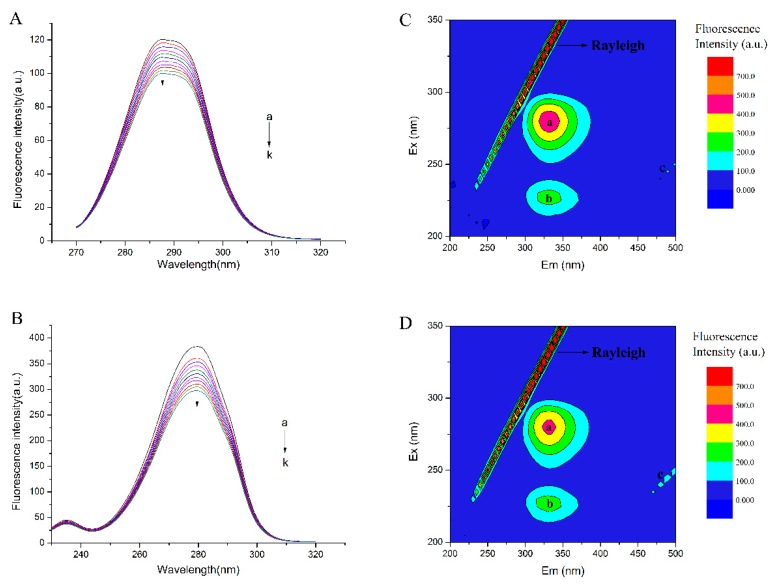
Synchronous fluorescence spectra of bLf with varying concentration of TCH (**A**: Δ*λ* = 15 nm, **B**: Δ*λ* = 60 nm). Fluorescence EEM landscapes of bLf (**C**) and the TCH-bLf systems (**D**).

The results were shown in Figure 4A,B. It was apparent in Figure 4A,B that the emission peaks almost had no change over the investigated concentration range. It indicated that TCH had little effect on the microenvironment of the tyrosine or tryptophan residues in bLf. Corresponding results showed that tyrosine residues in bLf had a typical absorption peaks around 287.4–287.8 nm (Figure 4A) while tryptophan had a typical absorption peaks around 279.4–279.6 nm (Figure 4B). In traditional and calibration mode, the synchronous fluorescence for tryptophan and tyrosine both reduced successively with the increase of TCH and fluorescence quenching also happened. The synchronous fluorescence for tryptophan and tyrosine also showed the consistent trends as that of bLf fluorescence. The fluorescence intensity of tyrosine residues decreased from 120 a.u. to 90 a.u., while the intensity of tryptophan decreased around 100 a.u. (from 370 a.u. to 270 a.u.). However, the emission peaks had little change over the investigated concentration range.

#### 2.6.3. Conformational Investigation by Fluorescence EEM Spectra

Fluorescence EEM spectroscopy is a rapid, selective, and sensitive technique. It has been proven to be a useful technique to provide detailed information about the conformational change of proteins [27]. In this paper, the data obtained from it was recorded in Figure 4C,D.

#### 2.6.4. Conformational Investigation by CD Spectra

**Figure 5:**
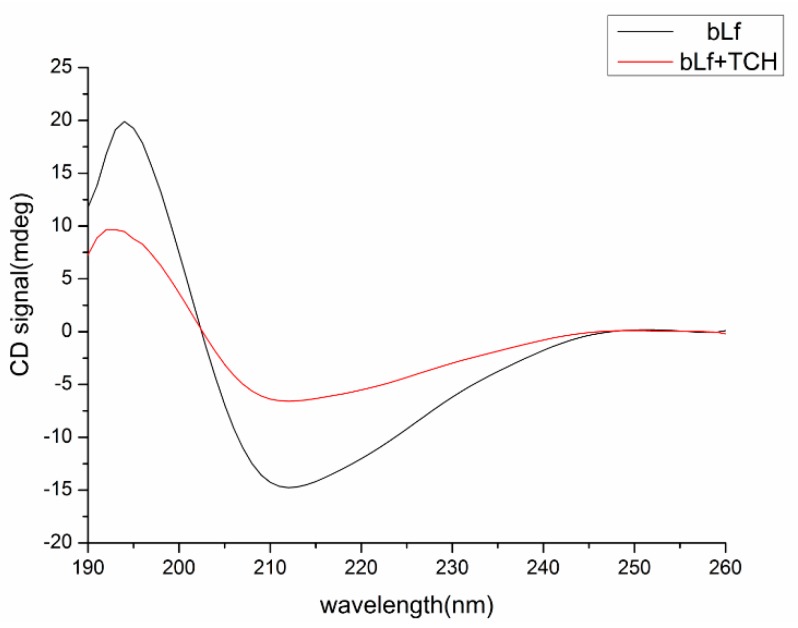
Far-UV CD spectra of bLf in the absence and presence of TCH at pH 6.6. c (TCH)/c (bLf) = 1:1.

To ascertain the possible influence of TCH binding on the secondary structure of bLf, CD measurement was performed (Figure 5). The CD spectra of bLf exhibited two negative bands in the ultraviolet region at about 211 nm and 240 nm, which is characteristic of the α-helix of proteins [28]. The results were expressed as mean residue ellipticity (*MRE*) in deg cm^2^ dmol^−1^ which is defined as:

(9) $$MRE\;=\;\frac{\theta_{obs}\left(mdeg\right)M}{nlC}$$

where *θ_obs_* is the CD in millidegrees, *M* is the molecular weight of the protein in g dmol^−1^, *n* is the number of amino acid residues, *l* is the path length (0.1 cm) of the cuvette, and *C* is the concentration of the protein in g L^−1^.

#### 2.6.5. Conformational Investigation by Molecular Modeling

**Figure 6:**
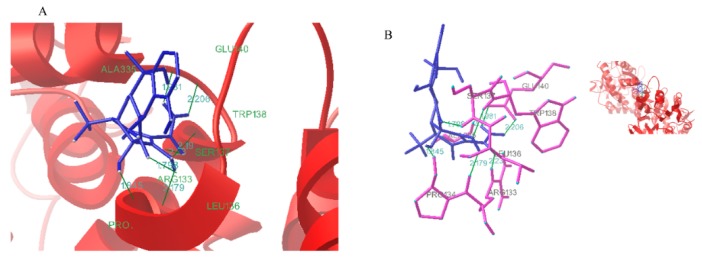
Molecular modeling study predicted orientation of the binding conformation of TCH with bLf. (**A**) Secondary structure of bLf around TCH. (**B**) Hydrogen bond between bLf with TCH.

The molecular modeling by the AutoDock 4.2 program has been employed to improve the understanding of the interaction of TCH with bLf. The best energy ranking results of the binding mode between TCH and bLf are shown in Figure 6 [19].

#### 2.6.6. ANS Surface Hydrophobicity Determination

ANS can bind with hydrophobic (non-polar) surfaces of proteins, leading to an increase in fluorescence intensity [29], hence it was used to determine the changes of hydrophobicity of bLf in this paper. Under conditions of fluorescent probes present in excess, protein relative fluorescence intensity *F* plotted against their concentration *C*, slope of the line is *S*_0_ [30]:

(10) $$S_{0}\;=\;\frac{\Delta F}{\Delta C}$$

**Figure 7:**
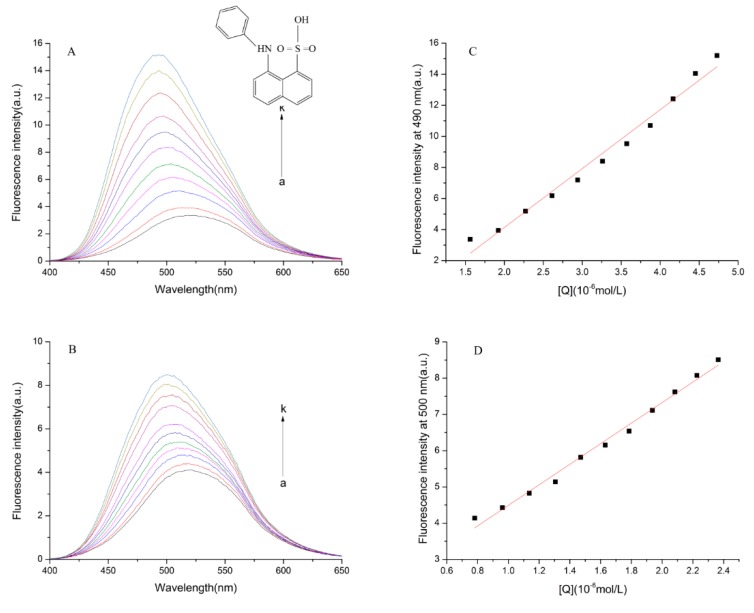
The structure of ANS. Fluorescence emission spectra of bLf-ANS system (**A**) and bLf-TCH-ANS system (**B**) with different concentration of bLf. Variation in relative fluorescence intensity of bLf-ANS (**C**) at 490 nm and bLf-TCH-ANS (**D**) at 500 nm with different concentration of bLf, c (TCH)/c (bLf) = 1:1.

Figure 7A,B revealed that the fluorescence intensity of ANS solution obviously increased with increasing bLf concentration. Furthermore, the fluorescence intensities of the bLf-TCH-ANS system were significantly lower than the bLf-ANS system.

### 2.7. Effect of TCH on the Interaction between bLf and Fe^3+^ Ion

**Figure 8:**
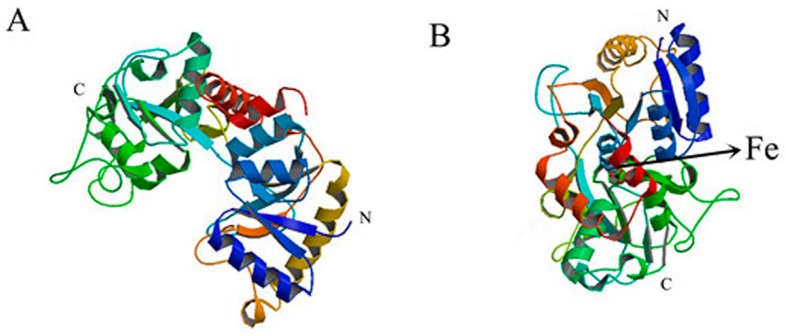

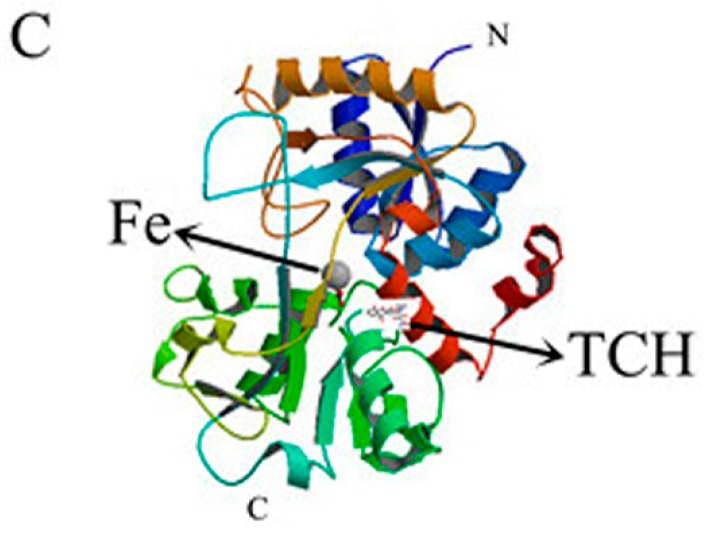
Spatial organizations of apo- and Fe-bLf (based on X-ray structural analysis).

**Figure 9:**
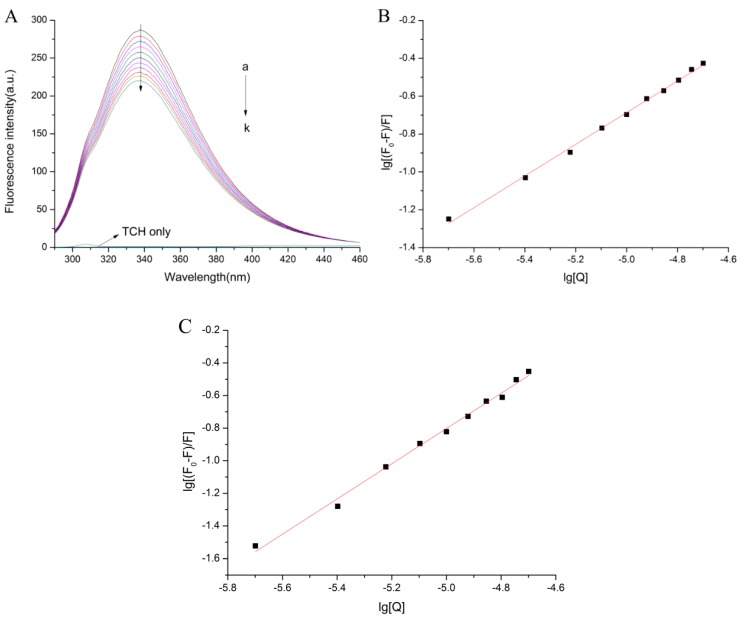
(**A**) The fluorescence quenching spectra about the impact of TCH on bLf and iron ion system (simulated the environment of bLf and iron in milk). Double-log Stern–Volmer plots for the quenching of bLf-Fe^3+^ in the absence (**B**) and presence (**C**) of TCH with different concentration of Fe^3+^ ion. c (TCH)/c (bLf) = 1:1.

Bovine lactoferrin (bLf) is an extremely polyfunctional iron-binding whey protein and its biological properties are believed to be due to its iron binding capacity. Antibiotic residues (TCH) in milk may affect the interaction between bLf and iron ions, which can affect the function of bLf. Spatial organizations of apo- and Fe-bLf (based on X-ray structural analysis) are shown in Figure 8. The average content of bLf in milk is about 1.3 g/L [6], and the content of iron in milk is 210 μg/L [31]. Thus, the ratio of bLf to iron in milk can be roughly calculated. We simulated the environment of bLf and iron in milk, Figure 9A shows the fluorescence quenching spectra regarding the effect of TCH on it.

## 3. Discussion

The ability of proteins to fluoresce is due to the presence of tryptophan (Trp), tyrosine (Tyr), and phenylalanine (Phe) residues. The phenomenon of fluorescence intensity of bLf quenched regularly with an increasing concentration of TCH showed that the interaction between TCH and bLf occurred, and the hydrophobic microenvironment of aromatic amino acid residues slightly changed [11].

The fluorescence quenching mechanism can be divided into radiation quenching and non-radiation quenching. Non-radiation quenching can be divided into dynamic quenching and static quenching. Static quenching mainly occurs between fluorophore and quenching agent through complexation. As the temperature increases, the stability of the compound decreased, so the static quenching constant will decrease. Although dynamic quenching is mainly caused by the collision and diffusion effect between the fluorophore and the quencher, the dynamic quenching constant will increase with the increase of temperature. Figure 1C and Table 1 show that the S–V equation of bLF shows a good linear relationship (straight line), indicating that the annihilation type is single, that is to say, only dynamic or static quenching occurred. It can be seen from Table 1 that as the temperature increases, the quenching constant *K_SV_* of bLf decreases to some extent, and the quenching rate constant *K_q_* is much larger than the maximum diffusion collision quenching rate constant (2.0 × 10^10^ L/mol/s) [17]. Therefore, it can be inferred that the quenching process belongs to static quenching, which means that TCH combines with bLf protein to form a complex.

From Table 2, we found that the number of binding sites *n* approximately equaled 1, indicating that there was one binding site. At the same time, according to the analysis, it is found that the binding constants *K_a_* and *K_SV_* of bLf and TCH decrease with the increase of temperature, indicating that the stability of the complex formed by TCH and bLf protein decreases with the increase of temperature. It is shown that the process of forming the complex is a typical exothermic reaction, which is the same as the change trend of the enthalpy change (∆*H*) in Table 2. The calculated thermodynamic parameters and *K_a_* values for the binding interaction between TCH and bLf were listed in Table 2. According to Ross’s research, when Δ*H* > 0, Δ*S* > 0, it is hydrophobic effects; when Δ*H* < 0, Δ*S* < 0, van der Waals forces and hydrogen bonds played an important role in the interaction; when Δ*H* < 0, Δ*S* > 0, electrostatic forces are the main force. In this work, the negative sign for free energy (Δ*G*) means that the binding process is spontaneous. Δ*H* < 0, Δ*S* > 0, indicated that electrostatic forces play major roles in the formation of a bLf-TCH complex [32].

According to Förster’s non-radiative energy transfer theory, there were three possible conditions for energy transfer energy [33]: the donor can produce fluorescence light; the absorption spectrum of the receptor overlaps enough with the donor’s fluorescence emission spectrum; and the distance between the donor and the acceptor is less than 8 nm. The energy transfer effect is related not only to the distance between the acceptor and the donor, but also to the critical energy transfer distance. It has been reported for bLf that *K*^2^ = 2/3, *n* = 1.336 and *Φ* = 0.118. Based on Figure 2A and Equations (5)–(7), the values obtained were *J* = 1.26 × 10^−15^ cm^3^ L mol^−1^, *R*_0_ = 1.74 nm, *r* = 2.81 nm and *E* = 0.053. The value of *r* (2.81 nm) was lower than 8 nm. These data were in line with conditions of Förster’s non-radiative energy transfer theory, indicated a non-radiative energy transfer from bLf to TCH occurred. Besides, the value of *r* was higher than the half *R*_0_ and less than two times *R*_0_, which again elucidated the quenching type belongs to static mode [19].

MST was utilized to further prove the interactions results by fluorescence spectroscopy. Through MST experiments, we could get the following results. Amplitude value was 8.87 (>7), the scattered distance between the scattered jumps and the curves is about 2, and the Amplitude values are more than 2–3 times the average distance, which showed that the fitting of the experimental data is reliable [34,35]. The *K_d_* value of bLf and TCH was 35,200 ± 8110 nM, the dissociation percentage of TCH and bLf was about 1.66% when the dissociation at equilibrium, which indicated that bLf has a strong affinity with TCH. The determined association constants obtained from MST experiments were basically consistent with the data obtained by fluorescence spectroscopy.

The conformational changes of bLf had been deduced by measuring the fluorescence intensities of protein amino acid residues without or with the addition of TCH. In order to confirm the changes of conformation, UV-vis absorption, synchronous fluorescence, fluorescence EEM, and CD spectra were further used. Finally, molecular docking was used to determine the most stable complexes.

The UV-vis absorption spectra clearly showed a new absorption peak appeared at 360 nm which suggested that the addition of TCH led to the conformation change of bLf. The maximum peak positions of the complexes had slight red shifts which showed that the hydrophobicity of the microenvironment of aromatic amino acid residues had decreased [36]. UV-vis spectra can also be used to determine the type of quenching. Figure 2B displayed palpable distinct changes between the absorption spectra of bLf after adding TCH. These results confirmed that the quenching process of bLf-TCH was static quenching because dynamic quenching only influenced the excited state of quenching molecule while it wouldn’t touch the absorption spectra of quenching substances [30]. This is consistent with the conclusion that protein fluorescence is judged by the fluorescence quenching mechanism.

The fluorescence intensity of tyrosine residues decreased between TCH and bLf.

The main cause of UV-visible absorption is the absorption of light by the amino acid side chain groups (Trp, Tyr, and Phe). In addition, peptide bonds also have a strong absorption of light. The peak at 280 nm in the ultraviolet-visible absorption spectrum is the absorption peak of the double bond of the Tyr and Trp residues. The peak appearing near 257 nm is the absorption peak of Phe, and the absorption peak of the main chain structure of the polypeptide appears at 210 nm. In general, the microenvironment near the amino acid residue is determined by the conformation of the protein molecule. If the conformation of the protein changes, the microenvironment and the ultraviolet-visible absorption spectrum near the amino acid residue will change. The reverse is also true, if there is a difference in the absorption spectra of proteins after the addition of small molecules, it can be said that the conformation of the protein has changed. The results were shown in Figure 4A,B, 120 a.u. to 90 a.u., while the intensity of tryptophan decreased around 100 a.u. (from 370 a.u. to 270 a.u.). The results showed that TCH did affect the microenvironment for Trp and Tyr, and the effect of tryptophan was greater than that of tyrosine.

Figure 4C,D and Table 4 present the contour plots and their characteristics of bLf and bLf-TCH complexes, respectively. As it was observed, the EEM landscapes showed four peaks: the highest peak was the Rayleigh scattering peak; peak A was related to the spectral characteristic of Trp and Tyr residues; peak B mainly revealed the fluorescence spectral behavior of polypeptide backbone structures, whose intensity was correlated with the secondary structure of protein; peak C was the second-order Rayleigh scattering peak. From Figure 4C,D, we found that the Rayleigh scattering peak of bLf increased after the addition of TCH. The fluorescence of peak A and peak B were quenched from 468.0, 247.5 to 431.1 and 239.5 with the addition of TCH. The results were consistent with the data of synchronous fluorescence, indicating that the conformation of bLf was indeed changed by TCH. Nevertheless, the mechanism for maintaining the stability of the bLf-TCH complex is still very complex, which requires further investigation.

Figure 5 showed that the intensity of the negative peak of bLf decreased with increasing concentration of TCH, which confirmed that TCH was bound to bLf. The fractions of α-helix, β-sheet, β-turn, and random coils were estimated by CDNN and shown in Table 5. The α-helical content of free and combined bLf was calculated to be 26.6% in free bLf and 20.9% in the bLf-TCH complex, its decline might be due to destruction of the hydrogen bonding networks of bLf by TCH. It suggested that the binding of TCH could result in the loosening of the structure of bLf. The increase of the random percentage indicated that the structure of bLf become a slightly looser than before [37,38]. Meanwhile, the content of β-sheets did not exhibit any obvious change. These results suggested that the interaction of TCH with bLf could affect the secondary structure of bLf, which may affect its physiological function.

As shown in Figure 6, a careful inspection of the binding site suggested the closer contact of hydroxyl groups of TCH with the tryptophan amino acid residue. The results indicated that three amino acid residues (Arg133; Ser137; Trp138) were involved in bLf binding with TCH [16]. Arg133 is a polar amino acid, so we speculate that Van der Waals forces may be involved in the interaction between protein and TCH. In addition, hydrogen bonds have appeared in the molecular docking diagram, indicating that there is hydrogen bonding in the interaction between protein and TCH. These results showed the binding of TCH to bLf is predominantly by hydrogen bonds and hydrophobic contacts within the hydrophobic core of proteins. By calculating the hydrophobicity, we found that *S*_0 (bLf-ANS)_ = 3.791 (Figure 7C) and *S*_0 (bLf-TCH-ANS)_ = 2.836 (Figure 7D). Therefore, we speculated that the addition of TCH not only altered the bLf structure but destroyed the original hydrophobicity, making it more likely to be exposed in aqueous solution. These results correlate well with our other conclusions.

The paramount structure of proteins is maintained by hydrophobic interactions. Change in the hydrophobicity or hydrophilicity of proteins change will directly alter the biological function of the protein. Speculate: protein precipitation will occur when the protein’s surface hydrophobicity increases. In contrast, the structures of proteins become loose and more likely to be exposed to the solution when the protein surface hydrophobicity decrease. As could be seen from Figure 7A,B, the fluorescence intensity of the different systems of protein obviously increased with increasing bLf concentration. Meanwhile, the fluorescence intensities of the bLf-ANS system after adding TCH were significantly lower than prior to joining TCH; we could speculate that TCH changed the structure and the original properties of the proteins. The values of the surface hydrophobicity are shown in Table 6, *S*_0 (bLf-ANS)_ = 3.791, *S*_0 (bLf-TCH-ANS)_ = 2.836. After joining ANS, the values of *S*_0_ decreased. This result indicated the polarity increased and the surrounding solution polarity changed. We speculate that TCH binding with bLf destroys the originally hydrophobic structure of the proteins, making it more likely to be exposed in aqueous solution. This conclusion needs to be further verified.

In order to study the impact on the interactions between bLf and Fe^3+^ ions in the presence of TCH, we calculated the binding constants (*K_a_* and *n*) between bLf and iron ions with and without TCH. The results are shown in Figure 9B,C. The calculated results were *K_a_* = 3.143 × 103 L/mol and *n* = 0.8368 without TCH. With TCH, the values obtained were *K_a_* = 3.996 × 104 L/mol and *n* = 1.081. The presence of TCH made the binding force larger and the number of binding sites also increased. Therefore, we can deduce that TCH altered the original structure of bLf and made its structure looser. This led to the iron binding sites being exposed and thus the binding forces between bLf and Fe^3+^ were heightened. This conjecture was verified by previous experiments. In addition, there was no site competition between TCH and Fe^3+^ (analysis could be shown by Figure 6 and Figure 8), which made the binding force larger. In a word, it will have a certain effect on protein function due to the numerous properties of bLf closely related to its iron binding capacity [10]. The specific impact on protein function also requires us to further explore.

## 4. Materials and Methods

### 4.1. Materials

The materials used in this project, including bovine lactoferrin (bLf, purity ≥85%, 80 kDa) from bovine whey, were purchased from the Sigma Chemical Company (St. Louis, MO, USA). Tetracycline hydrochloride (TCH, Mr: 480.90 Da) was bought from Aladdin Industrial Corporation (Shanghai, China). 8-Anilino-1-naphthalenesulfonic acid (ANS, purity ≥95%, Mr: 299.34 Da) was supplied by TCI chemicals Company (Tokyo, Japan). They were used without further purification. bLf was stored at 4 °C and TCH was stored −20 °C until use. All other reagents and solvents were of analytical reagent grade. Double distilled water was used in the whole experiment.

TCH and bLf were prepared with phosphate buffer solution (PBS) (0.05 M, pH 6.6) to get the stock solutions (1.0 × 10^−4^ mol/L), which would be diluted to obtain the different concentrations of working solutions with PBS buffer. Stock solution of 1.0 × 10^−3^ mol/L ANS was prepared in 0.1 M PBS buffer (pH 6.6). ANS stock solution was stored in a screw-capped container and wrapped in aluminum foil to avoid exposure to light. Metal ion stock solution preparation: FeCl_3_·6H_2_O was dissolved in double distilled water to get Fe^3+^ ion stock solution (1.0 × 10^−3^ mol/L). All solutions were used freshly after preparation and stored in a refrigerator at 4 °C.

### 4.2. Apparatus and Procedures

#### 4.2.1. Fluorescence Spectroscopy

The fluorescence data for the interaction between bLf and TCH was obtained by the F-7000 (Hitachi, Tokyo, Japan) fluorescence spectrophotometer. The working concentrations of bLf and TCH were 1.0 × 10^−6^ mol/L and 5.0 × 10^−5^ mol/L, respectively. With the addition of TCH, the final concentrations of TCH in samples was changed to 0, 0.5, 1.0, 1.5, 2.0, 2.5, 3.0, 3.5 4.0, 4.5, and 5.0 × 10^−6^ mol/L for TCH. The slit widths of excitation and emission were fixed at 5 nm. Moreover, 280 nm was set as excitation wavelength and the emission wavelength was obtained from 290 to 460 nm. The experiment was operated at three different temperatures (290 K, 300 K, and 310 K).

The scanned range of synchronous fluorescence spectra was from 200 to 360 nm. For the fluorescence EEM spectra, the emission and excitation wavelengths were obtained from 200 to 500 nm and 200 to 350 nm with 5 nm increments, respectively. Post-collection data were processed with MATLAB 8.3 [39,40]. All the synchronous fluorescence and fluorescence EEM spectra experiments were carried out at room temperature and pH 6.6.

#### 4.2.2. Microscale Thermophoresis (MST) Study

MST is a fast and accurate method for detecting ligand–protein interaction [32,33]. In this paper, the samples were detected by a Monolith NT.115 instrument, and the NT analysis software was used for data analysis to gain the *K_d_* values [41]. BLf and TCH were reconfigured with PBS buffer (pH 6.6), the concentration of bLf was 200 nM, and the certain addition of TCH was from 2 mM to 122.08 nM, respectively.

#### 4.2.3. UV-vis Absorption Spectroscopy

The UV-vis absorption spectra were obtained by the NanoDrop 2000c spectrometer (Thermo Fisher Scientific, Waltham, MA, USA) and recorded from 190 to 450 nm. In the experiment, 2.5 mL bLf solution was titrated by continuous additions of TCH. The final concentrations for TCH were 0, 1.0, 2.0, and 3.0 × 10^−6^ mol/L, respectively.

#### 4.2.4. CD Spectroscopy

For the CD spectroscopy experiment, a chirascan circular dichroism spectrometer (Applied PhotoPhysics, Surrey, United Kingdom) was used to analyze the structural change of bLf before and after the addition of TCH at far-UV (190–260 nm) regions. The concentration of bLf was 5.0 × 10^−5^ mol/L, adding a certain amount of TCH (1.0 × 10^−4^ mol/L), to give a final molar ratio of 1.0.

#### 4.2.5. Molecular Docking

For macromolecular docking, the bLf molecule model and TCH model were used for ligand docking. Docking simulations were performed using AutoDock 4.2 and the Lamarckian genetic algorithm was applied to calculate the possible conformation of the ligand molecule and macromolecule. The population size of the docking experiment was set as 60. The conformer with the lowest binding free energy was used for further analysis.

#### 4.2.6. ANS Surface Hydrophobicity Determination

A 3.0 mL portion of 1.0 × 10^−3^ mol/L solution of ANS was titrated by continuous additions of 1.0 × 10^−4^ mol/L bLf and bLf-TCH complex solutions (bLf:TCH = 1:1). After blending and standing in the dark reaction for 3 min, the fluorescence intensity was measured. The excitation and emission slit widths were fixed at 5 nm. The excitation wavelength was set at 380 nm and the emission wavelength was obtained from 390 to 650 nm.

#### 4.2.7. Impact of Fe^3+^ on the Interactions between TCH and bLf

To obtain a 1.0 cm quartz cell, the bLf and bLf-TCH (1:1) complex solutions were added to 2.5 ml and then Fe^3+^ ion was gradually added to them. Experiments were carried out at 300 K, pH 6.6. Other experimental parameters and conditions were the same as those for the fluorescence quenching spectra.

### 4.3. Statistical Analyses

All of the experiments were carried out in triplicate, with triplicate sample analyses being performed on each mixture. The results were presented as the means ± standard deviation. Statistical analysis was carried out using Origin 8.6 software All of the values were averaged and were plotted.

## 5. Conclusions

In this paper, the results obtained from experiments showed that TCH indeed interacted with bLf and caused a change of bLf structure. CD spectra further analyzed the transformation of the secondary component in the process of structural change and confirmed that the addition of TCH caused the protein structure to become loose. Additionally, fluorescence spectroscopy and UV-vis absorption spectra also revealed that the interaction process between TCH and bLf involved a static quenching mechanism and the main binding force was electrostatic. The results were further verified by molecular docking experiments. Finally, ANS surface hydrophobicity determination showed that the addition of TCH not only altered the bLf structure, but also destroyed the original hydrophobicity, leading to a decrease of bLf hydrophobicity. In summary, these studies provided accurate and comprehensive data for clarifying the binding mechanism of TCH with bLf. It also indicated that the presence of TCH could change the structure of the bLf, and thereby affect its own function. Experiments on surface hydrophobicity and research about the binding between bLf and iron ions further helped in understanding its effect on protein function. The results will be applicable in the milk industry and for food safety.

## Figures and Tables

**Table 1:** The quenching constants of the interaction between bLf and TCH.

Compound	*T* (K)	*K_sv_* (10^4^ L/mol)	*K_q_* (10^12^ L/mol/s)	R^2^
	290	6.88	6.88	0.9999
bLf + TCH	300	5.14	5.14	0.9997
	310	4.74	4.74	0.9999

**Table 2:** The thermodynamic binding parameters of the interaction between bLf and TCH at different temperatures.

Compound	*T* (K)	*K_a_* (10^4^ L/mol)	*n*	R^2^	△*H* (KJ/mol)	△*G* (KJ/mol)	△*S* (J/mol/K)
	290	5.154	0.978	0.9970		−107.99	
bLf + TCH	300	2.551	1.316	0.9765	−87.56	−108.70	70.45
	310	0.495	0.814	0.9932		−109.40	

**Table 3:** The binding parameters of TCH binding with bLf at 300 K.

Compound	*T* (K)	*K_a_* (10^4^ L/mol)	*E*	*n*	R (nm)	*R*_0_ (nm)
bLf + TCH	300	2.551	0.053	1.32	2.81	1.74

**Table 4:** TCH–bLf binding studied by MST.

Compound	Peak Position λex/λem (nm/nm)	Intensity *F*_0_	Peak Position λex/λem (nm/nm)	Intensity *F*_0_
bLf	280/332	468.0	225/331	239.5
bLf + TCH	280/332	431.1	225/331	247.5

**Table 5:** Secondary structure fractions of bLf complexes (CD spectra) with TCH.

Molar Ratio [bLf]/[TCH]	α-helix	β-sheet	Antiparallel	Parallel	Random Coil
1:0	26.6	18.3	16.1	10.0	36.3
1:1	20.9	19.6	28.1	11.7	40.7

**Table 6:** The values of the surface hydrophobicity of the bLf-ANS system and the bLf-TCH-ANS system.

Compound	*S*_0_	nm	R^2^
BLF + ANS	3.791	490	0.9908
BLF + ANS + TCH	2.836	500	0.9953

## Abbreviations

Tetracycline hydrochloride	TCH
Bovine lactoferrin	bLf
Phosphate buffer solution	PBS
8-Anilino-1-naphthalenesulfonic acid	ANS
MicroScale Thermophoresis	MST
Ultraviolet visible absorption spectra	UV-vis absorption spectra
Fluorescence Excitation-Emission Matrix spectra	fluorescence EEM spectra
Circular dichroism spectra	CD spectra
